# Supplementary material for: Timing of Dietary Fatty Acids to Optimize Reduced Risk of Type 2 Diabetes Mellitus: Findings from China Health and Nutrition Survey
Source: Nutrients. 2025 Jun 24;17(13):2089. doi: 10.3390/nu17132089 (PMC12250814; doi:10.3390/nu17132089)
Supplement: Supplementary file 1 [file nutrients-17-02089-s001.zip › nutrients-3575598-supplementary.pdf]

Online Supplemental Material

Table of Contents

Table S1. Baseline characteristics of subjects in the China Health and Nutrition Survey across categories of the difference in plant-sourced MUFAs intake between dinner and breakfast (*n* = 14,518)..........................2
Table S2. Multivariable-adjusted HRs (95% CIs) for the association of the differences in dietary fatty acids (SFAs, MUFAs, and PUFAs) intake between dinner and breakfast with the risk of T2D in the China Health and Nutrition Survey (1991–2015) (*n* = 14,518) 4
Table S3. Multivariable-adjusted HRs (95% CIs) for the association of the differences in subtypes of dietary fatty acids intake between dinner and breakfast with the risk of T2D in the China Health and Nutrition Survey (1991–2015) (*n* = 14,518)...5
Table S4. Sensitivity analysis of the HRs (95% CIs) for the association of the differences in subtypes of fatty acids intake between evening and forenoon with the risk of T2D that including snacks between each meal (*n* = 14,518)..6
Table S5. Sensitivity analysis of the HRs (95% CIs) for the association of the difference in dietary fatty acids intake between dinner and breakfast to the total consumption (Δ/sum) with the risk of T2D (*n* = 14,518) ...7
Table S6. Sensitivity analysis of the HRs (95% CIs) for the association of the differences in subtypes of fatty acids intake between dinner and breakfast with the risk of T2D after excluding subjects with extreme BMI (*n* = 12,300) 8
Table S7. Sensitivity analysis of the HRs (95% CIs) for the association of the differences in subtypes of fatty acids intake between dinner and breakfast with the risk of T2D after adjusting for AHEI (*n* = 14,518)........9
Table S8. Subgroup analysis of the HRs (95% CIs) for the association of the differences in subtypes of fatty acids intake between dinner and breakfast with the risk of T2D (*n* = 14,518) 10

Table S1. Baseline characteristics of subjects in the China Health and Nutrition Survey across categories of the difference in plant-sourced MUFAs intake between dinner and breakfast (n = 14,518)

	**Quintiles of plant-sourced MUFAs intake between dinner and breakfast**					**p Trend**
**Characteristics**	**Q1 [b]**	**Q2**	**Q3**	**Q4**	**Q5**	
Δ P-MUFAs (% en)	-6.4±0.275	-1.0±0.010	0.2±0.005	1.4±0.008	4.7±0.055	<0.001
n	2903	2904	2904	2904	2903	
Age (years)	43.6±0.3	43.4±0.3	43.7±0.3	43.4±0.3	44.6±0.3	0.028
Male (%)	48.2	46.9	48.1	48.2	43.7	0.002
Han (%)	93.7	90.9	86.9	85.7	86.4	<0.001
BMI (kg/m^2)	23.7±0.1	22.9±0.1	22.6±0.1	22.7±0.1	22.7±0.1	<0.001
Household income (yuan/yr)	39,033.6±832.6	27,900.7±597.2	23,102.5±511.8	27,993.6±742.5	30,340.5±702.4	<0.001
Urbanization index	72.2±0.3	58.5±0.4	53.5±0.4	58.1±0.4	64.5±0.4	<0.001
Married (%)	86.6	86.1	83.4	85.4	83.2	<0.001
≥middle school (%)	40.5	26.5	19.1	23.0	23.9	<0.001
Vigorous activity (%)	16.5	38.9	45.4	38.2	31.8	<0.001
Current smoker (%)	28.7	29.6	31.3	30.4	28.0	0.002
Alcohol drinker (%)	38.0	35.2	32.5	35.6	33.7	<0.001
History of hypertension (%)	15.4	14.8	13.3	14.3	13.7	0.152
Dietary intake						
Total energy (kcal/day)	2029.1±10.3	2158.1±10.0	2146.9±10.1	2161.6±9.4	2101.9±10.2	<0.001
Δ Energy	258.4±5.4	220.7±4.9	224.2±5.2	272.9±5.1	311.8±5.3	<0.001
Total carbohydrates (% en)	53.3±0.2	58.4±0.2	61.4±0.2	58.7±0.2	53.7±0.2	0.140
Δ Carbohydrates (% en)	-0.6±0.3	-6.0±0.2	-8.3±0.3	-12.5±0.2	-18.0±0.3	<0.001
Total protein (% en)	13.9±0.1	13.2±0.1	12.8±0.1	13.2±0.1	13.0±0.1	<0.001
Δ Protein (% en)	3.7±0.1	2.0±0.1	2.0±0.1	2.6±0.1	3.3±0.1	0.347
Total fat (% en)	32.7±0.2	28.4±0.2	25.7±0.2	28.1±0.2	33.3±0.2	0.154

Δ Fat (% en)	-3.3±0.3	4.4±0.2	7.5±0.2	11.0±0.2	16.8±0.2	<0.001
Total cereal (mg/day)	362.5±2.5	408.6±2.6	426.3±2.7	405.2±2.4	368.8±2.2	0.253
Total cholesterol (mg/day)	0.4±0.004	0.3±0.004	0.3±0.004	0.3±0.005	0.3±0.004	<0.001
Total P-MUFAs (% en)	8.3±0.1	6.9±0.1	5.2±0.1	6.9±0.1	10.8±0.1	<0.001
Total A-MUFAs (% en)	6.1±0.1	5.2±0.1	6.0±0.1	5.8±0.1	5.3±0.1	<0.001
Total SFAs (% en)	8.7±0.1	7.7±0.1	7.6±0.1	8.0±0.1	9.1±0.1	<0.001
Total PUFAs (% en)	10.3±0.1	9.1±0.1	7.5±0.1	8.1±0.1	9.1±0.1	<0.001
AHEI	38.6±0.1	40.4±0.1	40.1±0.1	39.4±0.1	38.2±0.1	<0.001

Data are means ± SE unless otherwise indicated. Individual income was inflated to 2015. Total energy intake was presented as energy density ($g \cdot 2000\ kcal^{-1} \cdot d^{-1}$), and the subtypes of fatty acids intake timing were expressed as the energy percentage of each separate meal (% en); Q, quintile; Δ, the difference in dietary components intake between dinner and breakfast (dinner – breakfast); AHEI, Alternative Healthy Eating Index.

Table S2. Multivariable-adjusted HRs (95% CIs) for the association of the differences in dietary fatty acids (SFAs, MUFAs, and PUFAs) intake between dinner and breakfast with the risk of T2D in the China Health and Nutrition Survey (1991–2015) (*n* = 14,518)

	Quintiles of dietary intake of fatty acids					
	**Q1**	**Q2**	**Q3**	**Q4**	**Q5**	***p* Trend**
**Δ SFAs intake between dinner and breakfast**						
Δ, range (% en)	< -0.16	-0.16-1.62	1.62-4.15	4.15-7.36	> 7.36	
Cases/n	201/2903	248/2904	230/2904	211/2904	158/2903	
HR (95% CI)	1.00	1.05 (0.87-1.27)	0.98 (0.80-1.19)	0.92 (0.75-1.14)	0.93 (0.73-1.17)	0.298
**Δ MUFAs intake between dinner and breakfast**						
Δ, range (% en)	< -0.58	-0.58-1.86	1.86-5.19	5.19-9.64	> 9.64	
Cases/n	229/2903	222/2904	239/2904	208/2904	150/2903	
HR (95% CI)	1.00	0.83 (0.69-1.00)	0.90 (0.75-1.08)	0.84 (0.68-1.02)	0.77 (0.61-0.97)	0.041
**Δ PUFAs intake between dinner and breakfast**						
Δ, range (% en)	< -0.75	-0.75-0.79	0.79-2.22	2.22-3.95	> 3.95	
Cases/n	223/2903	210/2904	220/2904	201/2904	194/2903	
HR (95% CI)	1.00	0.81 (0.67-0.99)	0.85 (0.70-1.03)	0.80 (0.66-0.98)	0.88 (0.72-1.08)	0.207

Δ, the difference in FAs intake between dinner and breakfast (dinner – breakfast). HRs (95% CIs) were estimated using time-dependent Cox proportional hazards models with adjustment for age, gender, ethnic, BMI, education, marital status, income, urbanization index, physical activity, smoking, drinking, history of hypertension, total energy intake, dietary cholesterol, cereal, carbohydrate and protein intake, lunch FAs intake and remaining subtypes of dietary FAs.

Table S3. Multivariable-adjusted HRs (95% CIs) for the association of the differences in subtypes of dietary fatty acids intake between dinner and breakfast with the risk of T2D in the China Health and Nutrition Survey (1991–2015) (*n* = 14,518)

	Quintiles of dietary intake of fatty acids					
	**Q1**	**Q2**	**Q3**	**Q4**	**Q5**	***p* Trend**
**Δ ALA intake between dinner and breakfast**						
Δ, range (% en)	< -0.16	-0.16-0.03	0.03-0.19	0.19-0.39	> 0.39	
Cases/n	228/2903	247/2904	190/2904	203/2904	180/2903	
HR (95% CI)	1.00	0.95 (0.79-1.15)	0.74 (0.60-0.90)	0.89 (0.73-1.08)	0.83 (0.67-1.02)	0.047
**Δ EPA intake between dinner and breakfast**						
Δ, range (% en)	< 0	0	0-0.0006	0.0006-0.008	> 0.008	
Cases/n	62/759	464/7560	29/392	271/2904	222/2903	
HR (95% CI)	1.00	1.19 (0.83-1.70)	0.91 (0.55-1.53)	0.93 (0.64-1.35)	0.91 (0.61-1.36)	0.199
**Δ DHA intake between dinner and breakfast**						
Δ, range (% en)	< 0	0	0-0.004	0.004-0.02	> 0.02	
Cases/n	56/585	495/8194	177/1913	180/1913	140/1913	
HR (95% CI)	1.00	0.66 (0.40-1.08)	0.63 (0.39-1.02)	0.54 (0.33-0.88)	0.44 (0.26-0.75)	0.004
**Δ PA intake between dinner and breakfast**						
Δ, range (% en)	< -0.001	-0.001-0.13	0.13-0.31	0.31-0.55	> 0.55	
Cases/n	205/2903	253/2904	223/2904	217/2904	150/2903	
HR (95% CI)	1.00	1.08 (0.87-1.33)	0.91 (0.70-1.17)	0.90 (0.67-1.19)	0.89 (0.63-1.26)	0.367
**Δ OA intake between dinner and breakfast**						
Δ, range (% en)	< -0.18	-0.18-2.13	2.13-5.16	5.16-9.16	> 9.16	
Cases/n	204/2903	236/2904	238/2904	211/2904	159/2903	
HR (95% CI)	1.00	0.99 (0.80-1.22)	1.03 (0.81-1.32)	1.03 (0.78-1.36)	1.01 (0.72-1.42)	0.880

Δ, the difference in FAs intake between dinner and breakfast (dinner – breakfast). HRs (95% CIs) were estimated using time-dependent Cox proportional hazards models with adjustment for age, gender, ethnic, BMI, education, marital status, income, urbanization index, physical activity, smoking, drinking, history of hypertension, total energy intake, dietary cholesterol, cereal, carbohydrate and protein intake, lunch FAs intake and remaining subtypes of dietary FAs.

Table S4. Sensitivity analysis of the HRs (95% CIs) for the association of the differences in subtypes of fatty acids intake between evening and forenoon with the risk of T2D that including snacks between each meal (n = 14,518)

	**Quintiles of dietary intake of fatty acids between evening and forenoon**					
	**Q1**	**Q2**	**Q3**	**Q4**	**Q5**	***p* Trend**
**Δ n-3 PUFAs intake between evening and forenoon**						
Δ, range (% en)	< -0.12	-0.12-0.07	0.07-0.23	0.23-0.44	> 0.44	
Cases/*n*	232/2903	228/2904	204/2904	209/2904	175/2904	
HR (95% CI)	1.00	0.93 (0.77-1.12)	0.82 (0.68-0.998)	0.92 (0.75-1.12)	0.79 (0.64-0.98)	0.048
**Δ n-6 PUFAs intake between evening and forenoon**						
Δ, range (% en)	< -0.34	-0.34-0.93	0.93-2.19	2.19-3.74	> 3.74	
Cases/*n*	204/2903	230/2904	210/2904	214/2904	190/2903	
HR (95% CI)	1.00	1.00 (0.83-1.21)	0.89 (0.73-1.09)	0.97 (0.80-1.19)	0.90 (0.73-1.11)	0.320
**Δ n-6/n-3 PUFAs intake between evening and forenoon**						
Δ, range (% en)	< -2.74	-2.74--0.15	-0.15-0.66	0.66-2.40	> 2.40	
Cases/*n*	152/2903	231/2904	220/2904	218/2904	127/2903	
HR (95% CI)	1.00	1.37 (1.08-1.74)	1.30 (1.01-1.67)	1.33 (1.04-1.69)	1.43 (1.15-1.77)	0.010
**Δ P-MUFAs intake between evening and forenoon**						
Δ, range (% en)	< -2.10	-2.10--0.22	-0.22-0.76	0.76-2.36	> 2.36	
Cases/*n*	217/2903	235/2904	210/2904	213/2904	173/2903	
HR (95% CI)	1.00	0.92 (0.76-1.12)	0.90 (0.74-1.10)	0.84 (0.69-1.03)	0.72 (0.58-0.90)	0.003
**Δ A-MUFAs intake between evening and forenoon**						
Δ, range (% en)	<0.00	0.00-1.63	1.63-4.76	4.76-8.67	> 8.67	
Cases/*n*	148/2233	302/3574	229/2904	203/2904	166/2903	
HR (95% CI)	1.00	1.19 (0.97-1.47)	1.09 (0.88-1.35)	0.94 (0.75-1.18)	1.12 (0.87-1.43)	0.843

Δ, the difference in FAs intake between evening and forenoon including snacks (evening – forenoon). HRs (95% CIs) were estimated using time-dependent Cox proportional hazards models with adjustment for age, gender, ethnic, BMI, education, marital status, income, urbanization index, physical activity, smoking, drinking, history of hypertension, total energy intake, dietary cholesterol, cereal, carbohydrate and protein intake, afternoon FAs intake (including snacks) and remaining subtypes of dietary FAs.

Table S5. Sensitivity analysis of the HRs (95% CIs) for the association of the difference in dietary fatty acids intake between dinner and breakfast to the total consumption (Δ/sum) with the risk of T2D (*n* = 14,518)

	Quintiles of dietary intake of fatty acids between dinner and breakfast					
	**Q1**	**Q2**	**Q3**	**Q4**	**Q5**	***p* Trend**
**Δ/ sum n-3 PUFAs intake between dinner and breakfast**						
Δ, range (% en)	< -0.19	-0.19-0.06	0.06-0.30	0.30-0.65	> 0.65	
Cases/*n*	220/2903	261/2904	220/2904	215/2904	132/2903	
HR (95% CI)	1.00	1.06 (0.88-1.28)	0.86 (0.70-1.04)	0.89 (0.73-1.08)	0.74 (0.58-0.93)	0.003
**Δ/sum n-6 PUFAs intake between dinner and breakfast**						
Δ, range (% en)	< -0.10	-0.10-0.10	0.10-0.30	0.30-0.57	> 0.57	
Cases/*n*	210/2903	225/2904	216/2904	233/2904	164/2903	
HR (95% CI)	1.00	0.87 (0.72-1.05)	0.81 (0.67-0.99)	0.97 (0.80-1.17)	0.91 (0.73-1.13)	0.652
**Δ/sum n-6/n-3 PUFAs intake between dinner and breakfast**						
Δ, range (% en)	< -0.29	-0.29--0.03	-0.03-0.10	0.10-0.31	> 0.31	
Cases/*n*	147/2903	228/2904	233/2904	230/2904	210/2903	
HR (95% CI)	1.00	1.29 (1.03-1.60)	1.30 (1.04-1.63)	1.30 (1.05-1.62)	1.43 (1.15-1.78)	0.005
**Δ/sum P-MUFAs intake between dinner and breakfast**						
Δ, range (% en)	< -0.35	-0.35--0.05	-0.05-0.13	0.13-0.35	> 0.35	
Cases/*n*	215/2903	2234/2904	233/2904	209/2904	157/2903	
HR (95% CI)	1.00	0.86 (0.71-1.05)	0.84 (0.68-1.02)	0.77 (0.63-0.95)	0.73 (0.58-0.91)	0.003
**Δ/sum A-MUFAs intake between dinner and breakfast**						
Δ, range (% en)	< 0.00	0.00-0.23	0.23-0.79	0.79-1.35	> 1.35	
Cases/*n*	162/2265	256/3542	227/2904	219/2904	184/2903	
HR (95% CI)	1.00	0.94 (0.76-1.17)	1.04 (0.84-1.28)	0.94 (0.76-1.17)	1.01 (0.81-1.25)	0.969

Δ/sum, the difference in FAs intake between dinner and breakfast (dinner – breakfast) to the total consumption. HRs (95% CIs) were estimated using time-dependent Cox proportional hazards models with adjustment for age, gender, ethnic, BMI, education, marital status, income, urbanization index, physical activity, smoking, drinking, history of hypertension, total energy intake, dietary cholesterol, cereal, carbohydrate and protein intake, lunch FAs intake and remaining subtypes of dietary FAs.

Table S6. Sensitivity analysis of the HRs (95% CIs) for the association of the differences in subtypes of fatty acids intake between dinner and breakfast with the risk of T2D after excluding subjects with extreme BMI (*n* = 12,300)

	Quintiles of dietary intake of fatty acids between dinner and breakfast					
	**Q1**	**Q2**	**Q3**	**Q4**	**Q5**	***p* Trend**
**Δ n-3 PUFAs intake between dinner and breakfast**						
Δ, range (% en)	< -0.16	-0.16-0.04	0.04-0.21	0.21-0.42	> 0.42	
Cases/*n*	197/2460	217/2460	171/2460	177/2460	154/2460	
HR (95% CI)	1.00	0.95 (0.78-1.16)	0.75 (0.61-0.93)	0.87 (0.70-1.07)	0.81 (0.65-1.01)	0.033
**Δ n-6 PUFAs intake between dinner and breakfast**						
Δ, range (% en)	< -0.69	-0.69-0.71	0.71-1.96	1.96-3.52	> 3.52	
Cases/*n*	193/2460	192/2460	186/2460	173/2460	172/2460	
HR (95% CI)	1.00	0.86 (0.70-1.06)	0.82 (0.66-1.00)	0.81 (0.65-1.00)	0.92 (0.74-1.13)	0.256
**Δ n-6/n-3 PUFAs intake between dinner and breakfast**						
Δ, range (% en)	< -2.57	-2.57--0.15	-0.15-0.70	0.70-2.43	> 2.43	
Cases/*n*	139/2460	204/2460	193/2460	192/2460	188/2460	
HR (95% CI)	1.00	1.38 (1.07-1.78)	1.25 (0.96-1.63)	1.36 (1.05-1.75)	1.31 (1.04-1.65)	0.085
**Δ P-MUFAs intake between dinner and breakfast**						
Δ, range (% en)	< -2.33	-2.33-0.30	0.30-0.68	0.68-2.24	> 2.24	
Cases/*n*	199/2460	192/2460	185/2460	188/2460	152/2460	
HR (95% CI)	1.00	0.85 (0.69-1.04)	0.86 (0.70-1.07)	0.81 (0.65-0.99)	0.68 (0.54-0.85)	0.002
**Δ A-MUFAs intake between dinner and breakfast**						
Δ, range (% en)	< 0.00	0.00-1.18	1.18-4.32	4.32-8.42	> 8.42	
Cases/*n*	152/2072	232/2848	191/2460	192/2460	149/2460	
HR (95% CI)	1.00	1.01 (0.81-1.26)	0.94 (0.75-1.17)	0.93 (0.74-1.17)	1.17 (0.91-1.50)	0.602

Δ, the difference in FAs intake between dinner and breakfast (dinner – breakfast). HRs (95% CIs) were estimated using time-dependent Cox proportional hazards models with adjustment for age, gender, ethnic, BMI, education, marital status, income, urbanization index, physical activity, smoking, drinking, history of hypertension, total energy intake, dietary cholesterol, cereal, carbohydrate and protein intake, lunch FAs intake and remaining subtypes of dietary FAs.

Table S7. Sensitivity analysis of the HRs (95% CIs) for the association of the differences in subtypes of fatty acids intake between dinner and breakfast with the risk of T2D after adjusting for AHEI (*n* = 14,518)

	Quintiles of dietary intake of fatty acids between dinner and breakfast					
	**Q1**	**Q2**	**Q3**	**Q4**	**Q5**	***p* Trend**
**Δ n-3 PUFAs intake between dinner and breakfast**						
Cases/*n*	233/2903	242/2904	188/2904	206/2904	179/2903	
HR (95% CI)	1.00	0.94 (0.78-1.13)	0.72 (0.59-0.88)	0.87 (0.72-1.06)	0.81 (0.66-1.00)	0.030
**Δ n-6 PUFAs intake between dinner and breakfast**						
Cases/*n*	222/2903	215/2904	208/2904	207/2904	196/2903	
HR (95% CI)	1.00	0.85 (0.70-1.03)	0.80 (0.66-0.97)	0.84 (0.69-1.03)	0.90 (0.74-1.10)	0.286
**Δ n-6/n-3 PUFAs intake between dinner and breakfast**						
Cases/*n*	153/2903	227/2904	221/2904	227/2904	220/2903	
HR (95% CI)	1.00	1.36 (1.07-1.73)	1.31 (1.02-1.68)	1.41 (1.11-1.79)	1.38 (1.12-1.71)	0.010
**Δ P-MUFAs intake between dinner and breakfast**						
Cases/*n*	230/2903	224/2904	206/2904	210/2904	178/2903	
HR (95% CI)	1.00	0.85 (0.71-1.04)	0.86 (0.70-1.05)	0.80 (0.66-0.98)	0.72 (0.58-0.89)	0.003
**Δ A-MUFAs intake between dinner and breakfast**						
Cases/*n*	165/2366	275/3441	218/2904	219/2904	171/2903	
HR (95% CI)	1.00	1.06 (0.86-1.31)	0.96 (0.78-1.18)	0.95 (0.77-1.18)	1.10 (0.86-1.39)	0.904

AHEI=Alternative Healthy Eating Index. Δ, the difference in FAs intake between dinner and breakfast (dinner – breakfast). HRs (95% CIs) were estimated using time-dependent Cox proportional hazards models with adjustment for age, gender, ethnic, BMI, education, marital status, income, urbanization index, physical activity, smoking, drinking, history of hypertension, total energy intake, dietary cholesterol, cereal, carbohydrate and protein intake, lunch FAs intake, remaining subtypes of dietary FAs and AHEI.

Table S8. Subgroup analysis of the HRs (95% CIs) for the association of the differences in subtypes of fatty acids intake between dinner and breakfast with the risk of T2D (n=14,518)

		Quintiles of dietary intake of fatty acids between dinner and breakfast						
		Q1	Q2	Q3	Q4	Q5	*p* Trend	*p* Interaction
**Δ n-3 PUFAs intake between dinner and breakfast**								
Aged ≤ 40	Cases/*n*	51/1349	62/1349	48/1313	52/1339	37/1153		0.128
	HR (95% CI)	1.00	1.09 (0.74-1.61)	0.97 (0.64-1.47)	1.16 (0.76-1.75)	1.03 (0.65-1.64)	0.795	
Aged > 40	Cases/*n*	182/1554	180/1555	140/1591	154/1565	142/1750		
	HR (95% CI)	1.00	0.92 (0.74-1.15)	0.69 (0.54-0.86)	0.84 (0.67-1.05)	0.77 (0.61-0.98)	0.018	
**Δ n-6 PUFAs intake between dinner and breakfast**								
Aged ≤ 40	Cases/*n*	43/1302	49/1318	72/1374	50/1314	36/1195		0.627
	HR (95% CI)	1.00	1.05 (0.69-1.61)	1.46 (0.99-2.15)	1.10 (0.72-1.68)	0.98 (0.62-1.56)	0.874	
Aged > 40	Cases/*n*	179/1601	166/1586	136/1530	157/1590	160/1708		
	HR (95% CI)	1.00	0.81 (0.65-1.01)	0.66 (0.53-0.83)	0.78 (0.62-0.97)	0.89 (0.71-1.12)	0.205	
**Δ n-6/n-3 PUFAs intake between dinner and breakfast**								
Aged ≤ 40	Cases/*n*	42/1288	46/1195	51/1335	57/1334	54/1351		< 0.001
	HR (95% CI)	1.00	1.06 (0.65-1.73)	1.02 (0.62-1.67)	1.13 (0.70-1.81)	1.08 (0.70-1.65)	0.671	
Aged > 40	Cases/*n*	111/1615	181/1709	170/1569	170/1570	166/1552		
	HR (95% CI)	1.00	1.44 (1.09-1.90)	1.39 (1.04-1.85)	1.49 (1.12-1.96)	1.45 (1.13-1.87)	0.012	
**Δ P-MUFAs intake between dinner and breakfast**								
Aged ≤ 40	Cases/*n*	58/1313	58/1318	48/1301	45/1314	41/1257		0.031
	HR (95% CI)	1.00	0.90 (0.60-1.33)	0.86 (0.57-1.32)	0.74 (0.48-1.13)	0.81 (0.51-1.26)	0.201	
Aged > 40	Cases/*n*	172/1590	166/1586	158/1603	165/1590	137/1646		
	HR (95% CI)	1.00	0.84 (0.67-1.05)	0.86 (0.68-1.09)	0.81 (0.65-1.02)	0.70 (0.55-0.90)	0.010	
**Δ A-MUFAs intake between dinner and breakfast**								
Aged ≤ 40	Cases/*n*	36/1037	62/1590	60/1336	62/1334	30/1206		0.323
	HR (95% CI)	1.00	0.92 (0.58-1.46)	0.95 (0.62-1.46)	1.08 (0.70-1.67)	0.80 (0.47-1.36)	0.773	
Aged > 40	Cases/*n*	129/1329	213/1851	158/1568	157/1570	141/1697		
	HR (95% CI)	1.00	1.07 (0.85-1.37)	0.93 (0.73-1.19)	0.89 (0.70-1.14)	1.10 (0.84-1.45)	0.914	

Δ, the difference in FAs intake between dinner and breakfast (dinner – breakfast). HRs (95% CIs) were estimated using time-dependent Cox proportional hazards models with adjustment for age, gender, ethnic, BMI, education, marital status, income, urbanization index, physical activity, smoking, drinking, history of hypertension, total energy intake, dietary cholesterol, cereal, carbohydrate and protein intake, lunch FAs intake, remaining subtypes of dietary FAs.
